# Supplementary material for: Delivery of Oligonucleotides Using a Self-Degradable Lipid-Like Material
Source: Pharmaceutics. 2021 Apr 13;13(4):544. doi: 10.3390/pharmaceutics13040544 (PMC8070490; doi:10.3390/pharmaceutics13040544)
Supplement: Supplementary file 1 [file pharmaceutics-13-00544-s001.pdf]

pharmaceutics

Supplementary Material: Delivery of Oligonucleotides Using a Self-Degradable Lipid-Like Material

Hiroki Tanaka, Nae Takata, Yu Sakurai, Tokuyuki Yoshida, Takao Inoue, Shinya Tamagawa, Yuta Nakai, Kota Tange, Hiroki Yoshioka, Masatoshi Maeki, Manabu Tokeshi and Hidetaka Akita

Table S1. Detailed list of supplier's information of the reagents.

Reagent	Manufacturer	Product number
ssPalmO-Phe	NOF CORPORATION	COATSOME® SS-OP
ssPalmO-P4C2	NOF CORPORATION	COATSOME® SS-OC
ssPalmE-P4C2	NOF CORPORATION	COATSOME® SS-EC
Dioleoyl-sn-glycero-pshophatidyl choline (DOPC)	NOF CORPORATION	COATSOME® MC-8181
Distearoyl-sn-glycero-pshophatidyl choline (DSPC)	NOF CORPORATION	COATSOME® MC-8080
1-(Monomethoxy polyethyleneglycol2000)2,3-dimyristoylglycerol (DMG-PEG2000)	NOF CORPORATION	SUNBRIGHT® GM-020
Phosphate buffered saline	NACALAI TESQUE	11482-15
DL-Malic acid	NACALAI TESQUE	21029-55
Citric acid, anhydrous	NACALAI TESQUE	09109-85
Tris(hydroxymethyl)aminomethane	NACALAI TESQUE	35406-75
Sodium chloride	NACALAI TESQUE	31320-05
Sodium hydroxide	NACALAI TESQUE	06338-75
Ethanol 99.5%	NACALAI TESQUE	14712-05
UltrapureTM distilled water	Thermo Fisher Scientific	10977-023
Quant-IT™ RiboGreen™ RNA assay kit	Thermo Fisher Scientific	R11490
Quant-IT™ OliGreen™ ssDNA assay kit	Thermo Fisher Scientific	O11492
Cholesterol	SIGMA	C3045-5G
TNS	SIGMA	T9792-250MG
Amicon Ultra-4-100K	Merck	UFC810096
Amicon Ultra-15-100K	Merck	UFC910096
RNAlater Stabilization Solution	QIAGEN	76104
RNeasy Mini Kit	QIAGEN	74104
BCA protein Assay Kit	TAKARA	T9300A
One Step TB Green® PrimeScript™ PLUS RT-PCR Kit (Perfect Real Time)	TAKARA	PR096A
Otsuka normal Saline	Otsuka Pharmaceutical	0815
Hydrogen chloride	Wako	080-01066
Heparin sodium 5,000 Units/ 5mL	MOCHIDA	224122458
BIOPHENTM FVII Kit	Hyphen Biomed	221304

COATSOME and SUNBRIGHT are registered trademarks of NOF CORPORATION in Japan and other countries.

Table S2. Sequences of antisense oligonucleotides and primers.

Name	Sequence
ASOap	GCattggtatTCA
ASOgr	GTCtctttaccTGG
Gapdh forward primer	gtgtgaacggatttggccgt
Gapdh reverse primer	gacaagcttcccattctcgg
ApoB forward primer	ccaagagagtgacatggcgtta
ApoB reverse primer	tggaggccactttttggtatc
Nr3c1 forward primer	actgtccagcatgccgctat
Nr3c1 reverse primer	gcagtggcttgctgaattcc

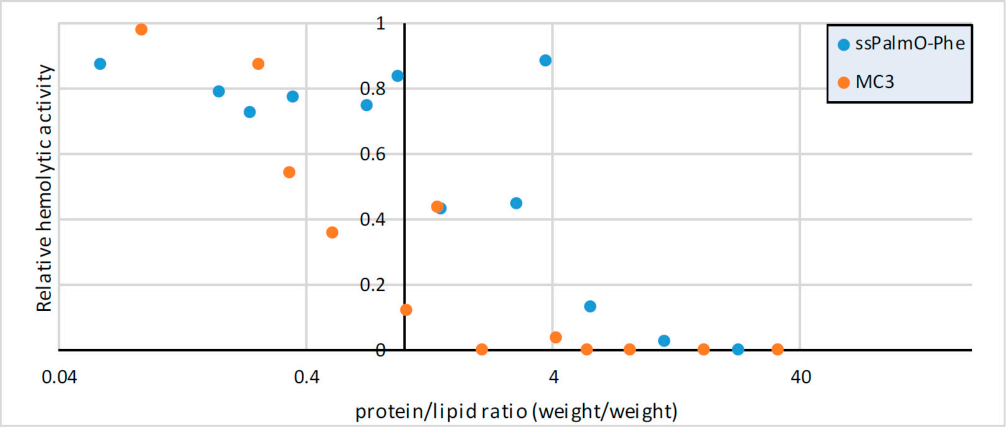

Figure S1. Scattered plot of relative protein amount-relative hemolysis.

LNPs containing ssPalmO-Phe or DLin-MC3-DMA (MC3) were prepared by the method described in the main text. The lipid composition was ssPalmO-Phe or MC3/DSPC/cholesterol/ DMG-PEG2000 = 50/10/38.5/1.5. Hemolytic activity was assessed by changing the total lipid concentration from 1.56 μM to 400 μM at different amounts of serum protein (20–120 μg/mL). Individual data in Figure 7 were then plotted. Relative protein amount against lipids (protein/lipid ratio) was calculated from the molecular weight of the lipids. The hemolytic activity was normalized by the values obtained for serum-free samples. Since the lipid concentration of 1.56 μM did not show significant hemolytic activity, even in the absence of proteins, the concentration was omitted from the analysis.

The relative hemolytic activity gradually decreased as the protein/lipid ratio increased. The relative hemolytic activity of the MC3 decrease at smaller protein/lipid ratio than ssPalmO-Phe. From this result, the serum protein resistance of ssPalmO-Phe was concluded.

Citation: Tanaka, H.; Takata, N.; Sakurai, Y.; Yoshida, T.; Inoue, T.; Tamagawa, S.; Nakai, Y.; Tange, K.; Yoshioka, H.; Maeki, M.; et al. Delivery of oligonucleotides using a self-degradable lipid-like material. *Pharmaceutics* **2021**, *12*, x. https://doi.org/10.3390/10.3390/pharmaceutics13040544

Publisher's Note: MDPI stays neutral with regard to jurisdictional claims in published maps and institutional affiliations.

Copyright: © 2021 by the authors. Submitted for possible open access publication under the terms and conditions of the Creative Commons Attribution (CC BY) license (http://creativecommons.org/licenses/by/4.0/).

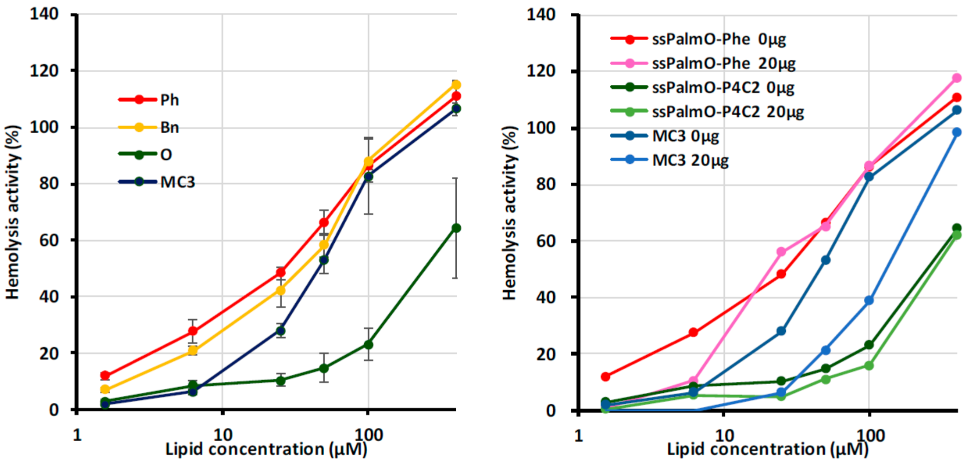

Figure S2. Hemolytic activity in the presence of the serum proteins.

LNPs containing the ssPalmO-Phe, ssPalmO-Ben, ssPalmO-P4C2, and DLin-MC3-DMA (MC3) was prepared by the method described in the main text. The lipid composition for the LNP$_{\text{ssPalm}}$ was ssPalm/cholesterol/DMG-PEG2000 = 70/30/1.5. The lipid composition for the LNP$_{\text{MC3}}$ was MC3/DSPC/cholesterol/ DMG-PEG2000 = 50/10/38.5/1.5. The dose-response curve of the hemolytic activity was assessed by changing the total lipid concentration from 1.56 μM to 400 μM. In the case of a hemolysis assay in the presence of the serum protein, the serum was collected and the protein concentration was quantified by means of a BCA Protein Assay kit (TAKARA) according to the manufacture's protocol. The serum (20 μg of protein) was added to the red blood cells and the hemolysis assay was conducted.

The hemolytic activity of the optimal lipid composition for siRNA (ssPalm/cholesterol = 70/30) was evaluated. Dose-dependency was clearly observed (**Left figure**). The hemolytic activity of the LNP$_{\text{ssPalmO-Phe}}$, LNP$_{\text{ssPalmO-Ben}}$, and LNP$_{\text{MC3}}$ was higher than that of the LNP$_{\text{ssPalmO-P4C2}}$. In the range of low lipid concentration (1.56-20 μM), the LNP$_{\text{ssPalmO-Phe}}$ and the LNP$_{\text{ssPalmO-Ben}}$ showed a higher hemolytic activity than the LNP$_{\text{MC3}}$. In the presence of 20 μg of serum proteins, the hemolytic activity of the LNP$_{\text{MC3}}$ was attenuated (**Right figure**). On the other hand, the hemolytic activity of the LNP$_{\text{ssPalmO-Phe}}$ was not affected by serum proteins. The LNP$_{\text{ssPlamO-P4C2}}$ also showed resistance against the serum proteins although the original hemolytic activity was lower than that of the LNP$_{\text{ssPalmO-Phe}}$ and LNP$_{\text{MC3}}$. From these observations, it was concluded that the chemical structure of the ssPalm appeared to confer resistance against the serum proteins to the LNP.
